# Cervicovaginal lavage fluid iron and manganese levels in women with and without vaginitis

**DOI:** 10.1186/s12905-026-04339-9

**Published:** 2026-02-24

**Authors:** Barbara Kozma, David Ratonyi, Bence Kozma, Peter Takacs, Attila G. Sipos

**Affiliations:** 1 Faculty of Medicine, Department of Obstetrics and Gynecology, University of Debrecen, Nagyerdei krt. 98, Debrecen, 4032 Hungary; 2 Doctoral School of Nutrition and Food Sciences, University of Debrecen, Egyetem tér 1, Debrecen, 4032 Hungary; 3 Department of Obstetrics and Gynecology, Division of Female Pelvic Medicine and Reconstructive Surgery, Eastern Virginia Medical School, 825 Fairfax Avenue, Suite 526, Norfolk, VA 23507-2007 USA

**Keywords:** Vaginitis1, Cervicovaginal lavage (CVL)2, Iron3, Manganese4, Trace metal homeostasis5

## Abstract

Transition metals such as iron and manganese are critical for cellular metabolism, redox balance, and immune function, and they also influence host–pathogen interactions. Vaginitis is characterized by mucosal inflammation that may alter local trace-element homeostasis. We hypothesize that inflammatory conditions such as vaginitis may be associated with altered cervicovaginal iron and manganese levels. We performed a prospective, single-center cohort study at the University of Debrecen (Hungary) between April 2023 and April 2024, enrolling 80 women ≥ 18 years with vulvovaginal complaints. Participants were assessed at baseline and at weeks 4, 8, and 12 with clinical evaluation and cervicovaginal lavage (CVL) sampling, collected using metal-free instruments. Participants were randomized 1:1 to receive either fluconazole alone or a single baseline oral fluconazole plus an intravaginal zinc-containing hydrogel. CVL samples were digested and analyzed for iron and manganese using ICP-OES. Group comparisons were performed using linear mixed-effects models with a random intercept for participant. CVL Fe concentrations were higher in women with culture-positive vaginitis compared with controls (mean ± SD: 56.36 ± 24.84 vs. 44.64 ± 20.11), with a significant between-group difference in mixed-effects models (Δ = 8.82, 95% CI 0.74 to 16.89; *p* = 0.032). Mn concentrations did not differ between vaginitis and controls (1.42 ± 0.73 vs. 1.09 ± 0.77; *p* = 0.542). In subgroup analyses, Fe remained significantly higher in vaginitis compared with controls among women who did not use the hydrogel (60.69 ± 26.53 vs. 47.27 ± 21.87; *p* = 0.041), whereas the difference was attenuated and no longer significant among hydrogel users (53.64 ± 23.62 vs. 41.75 ± 17.71; *p* = 0.148). Within the vaginitis cohort, Fe and Mn concentrations were similar across culture-defined phenotypes (bacterial-positive vs. fungal-positive) and between fungal culture-negative and fungal-positive samples. Culture-positive vaginitis was associated with higher total cervicovaginal iron concentrations, while manganese remained largely unchanged. Adjunct topical zinc therapy appeared to temper the vaginitis-associated elevation in iron, consistent with improved mucosal integrity and reduced inflammation-driven iron availability. Total Fe and Mn concentrations in CVL did not distinguish vaginitis phenotypes, supporting their use primarily as reference values and motivating future work focused on bioavailable metal fractions and metal-binding host proteins. The study protocol was approved by the Hungarian National Institutional Review Medical Research Council (approval No. 3282-7/2023/EUIG).

## Introduction

Trace elements such as iron, zinc, manganese, and copper are indispensable for life due to their fundamental roles as structural and catalytic cofactors in a diverse array of proteins. These metals are integral to numerous biochemical processes, including enzymatic reactions, electron transport, and regulatory functions, thereby ensuring proper cellular function and organismal viability [1]. Approximately 50% of all enzymes require the association with a specific metal ion to achieve and maintain their catalytic activity [2]. Given these central roles, trace metals in their ionic forms are essential for sustaining life, acting as cofactors that enable protein function, regulate enzymatic activity, and stabilize protein structure [3]. Iron is an essential cofactor for metabolic processes, including host and pathogen respiration. During infection, the host restricts iron availability to limit microbial growth, while pathogens evolve strategies to acquire it within host-cell niches. During inflammation, upregulation of the iron-regulatory hormone hepcidin leads to a further reduction in serum iron levels, whereas hepcidin deficiency increases host susceptibility to bacterial infections, highlighting the central role of iron in host–pathogen interactions [4]. Thus, iron not only supports host physiology but also shapes antimicrobial defense and pathogen adaptation [5]. Reactive oxygen species (ROS) are closely linked to iron, which regulates their production. At controlled levels, ROS serve as key signaling molecules in immunity, cell proliferation, and apoptosis, whereas excessive ROS cause oxidative stress and cellular damage. Both hosts and pathogens modulate iron availability to influence ROS for their benefit. The pathogen-specific nature of such regulatory mechanisms renders them promising targets for therapeutic intervention [6]. Manganese is an essential trace element that supports both innate and adaptive immunity, primarily as a cofactor for key metalloenzymes such as mitochondrial manganese superoxide dismutase (MnSOD), which mitigates oxidative stress. It is also critical for enzymatic pathways regulating inflammation, cell signaling, and pathogen defense [7]. For successful pathogenesis, bacteria must adapt to and actively respond to the dynamic conditions within the host, including variations in manganese availability [8]. Although it is unclear under what conditions invaders might encounter elevated manganese levels, these findings underscore the wide range of metal environments pathogens face within the host. It has been suggested that controlling intracellular manganese levels is essential for maintaining the proper manganese-to-iron ratio within the cell [2, 9]. Vaginitis may result from infectious agents, inflammatory processes, or disruptions in the normal vaginal microbiota. Clinical manifestations commonly include malodor, irritation, burning sensation, pruritus, dysuria, dyspareunia, and alterations in the characteristics or volume of vaginal discharge [10, 11]. The human vaginal microbiota consists of a complex community of commensal microorganisms and opportunistic pathogens that colonize and interact within the vaginal microenvironment [12]. The vaginal microbiome constitutes a dynamic microbial ecosystem essential for maintaining vaginal health, and its composition varies across the female lifespan, influenced by hormonally driven changes occurring in prepubertal, reproductive, and postmenopausal stages [13]. Vaginitis is frequently associated with the excessive proliferation of yeasts, predominantly Candida albicans, which under normal conditions constitute a natural component of the vaginal microbiota [14, 15]. Risk factors predisposing to vaginitis include age, multiple sexual partnerships, limited use of barrier contraceptive methods, menstruation, history of abortion, and inadequate personal hygiene [12]. The management of vaginitis is etiology-dependent. The therapeutic goals are eradication of the causative agent, restoration of the normal vaginal microbiota, and relief of symptoms. Our hypothesis is that inflammatory processes, including vaginitis, are associated with alterations in the concentrations of iron and manganese in the cervicovaginal lavage fluid.

## Materials and methods

We conducted a prospective, single-center longitudinal study embedded within an ongoing randomized clinical trial at the outpatient clinic of the Department of Obstetrics and Gynecology, University of Debrecen (Hungary). This report presents a preliminary, exploratory analysis of consecutively enrolled participants assessed between April 2023 and April 2024. The study protocol was approved by the Hungarian National Institutional Review Medical Research Council (approval No. 3282-7/2023/EUIG), and all participants provided written informed consent prior to any study procedure. Women aged ≥ 18 years presenting with vulvovaginal complaints such as discharge, odor, itching, pain, dysuria, skin irritation, burning, dyspareunia, or related discomfort were eligible for enrolment. Exclusion criteria included pregnancy, breastfeeding, abnormal cervical cytology within the prior year, gynecologic malignancy, relevant systemic diseases, known azole hypersensitivity, liver disease, recent systemic or topical antifungal therapy or systemic antibiotic use within a predefined window, and any laboratory-confirmed sexually transmitted infection. A total of eighty symptomatic women were enrolled in the parent trial and were evaluated at four time points: baseline (week 0), week 4, week 8, and week 12. At each visit, demographic and clinical characteristics and interval history were recorded, including age, BMI, parity, history of recurrent infections, and interim treatments. Vaginitis status was assessed at each visit by an experienced obstetrician–gynecologist based on symptom assessment, gynecologic examination, and routine vaginal culture processed according to institutional standards. When a vaginal yeast infection was identified, participants received oral antifungal therapy per local practice, and additional therapies were initiated for other vaginal conditions if clinically indicated; interim treatments were documented at follow-up visits. Standard diagnostic criteria for bacterial vaginosis such as Amsel criteria, Nugent scoring, pH testing, or NAAT-based panels were not used in this study, and therefore microbiological phenotypes are reported as culture-defined categories rather than BV. Gynecologic examinations were performed using a plastic speculum and plastic instruments to minimize exogenous metal contamination while assessing clinical signs including discharge and irritation. After symptom assessment, cervicovaginal lavage (CVL) was collected at each visit for iron and manganese quantification. CVL collection involved instilling 10 mL sterile normal saline onto the vaginal walls and cervix, maintaining contact for 60 s, followed by three successive aspirations and re-instillations using a plastic syringe; the pooled fluid was finally aspirated from the posterior fornix. Samples were aliquoted and stored at − 80 °C until elemental analysis. Participants in the symptomatic cohort were randomized in a 1:1 ratio as part of the ongoing randomized clinical trial (ClinicalTrials.gov identifier NCT05895162; registration date: 2023-06-08). The control arm received standard treatment with a single oral dose of fluconazole at baseline, whereas the intervention arm received fluconazole followed by intravaginal JUVIA zinc-containing hydrogel (Fempharma LLC, Debrecen, Hungary). Participants were instructed to self-administer 2 mL of vaginal gel using the provided applicator for 12 weeks, daily during the first 2 weeks and then twice weekly for the subsequent 10 weeks; adherence was tracked via participant logs, and adverse events were queried and documented at each visit. For analysis, we applied a priori operational definitions using the Vulvovaginal Symptoms Questionnaire (VSQ-21) [16] and a Candida Severity Score (CSS) [17], in combination with routine culture results. Asymptomatic controls were defined as individuals with VSQ-21 = 0, CSS < 3, and negative vaginal culture, while the symptomatic culture-positive vaginitis cohort was defined as CSS ≥ 3 and VSQ-21 > 0 with a positive vaginal culture showing growth of yeast and/or bacteria. Within the symptomatic cohort, culture-defined phenotypes were based on microbiology at the relevant visit, with fungal-positive defined by yeast growth detected on culture (with or without bacterial growth), bacterial-positive defined by bacterial growth in the absence of yeast, and culture-negative symptomatic defined by the presence of symptoms with a negative routine culture. For elemental analysis, 5.00 mL of each CVL specimen was transferred using an automatic pipette into glass beakers, the aqueous fraction was evaporated on an electric heating plate, and the residue underwent wet digestion using 3 mL 65% HNO₃ (Sigma-Aldrich/Merck, Budapest, Hungary) and 1 mL 30% H₂O₂ (VWR). Digests were quantitatively transferred into polypropylene centrifuge tubes, brought to 12 mL with ultrapure water (Synergy UV, Millipore), and filtered through 0.45 μm PVDF membrane filters. Elemental concentrations were determined by inductively coupled plasma optical emission spectrometry (ICP-OES; Agilent 5110 Vertical Dual View, Santa Clara, CA, USA) equipped with an Agilent SPS4 autosampler, Meinhard^®^ nebulizer, and double-pass spray chamber. Calibration used a seven-point matrix-matched series prepared from a certified multi-element stock solution (1000 mg/L, ICP IV, Certipur, Merck) and analytical-grade NaCl in 0.1 M HNO₃, and instrumental measurements were performed in triplicate with the mean value used for statistical analyses. Because no certified reference material is available for this specific sample matrix, analytical accuracy was evaluated using matrix-matched independent standard recovery testing with an acceptance window of ± 5%, and procedural blanks were included and processed identically to clinical samples to monitor reagent background and minimize contamination. During the measurements the limits of detection (LoD) were 1.35 µg/L for Cu, 6.08 µg/L for Fe, 0.30 µg/L for Mn, and 7.26 µg/L for Zn, while the limits of quantification (LoQ) were 4.51 µg/L for Cu, 20.3 µg/L for Fe, 1.01 µg/L for Mn, and 24.1 µg/L for Zn. The operating parameters used for ICP-OES analysis and element-specific limits of detection and quantification are provided in Tables 1 and 2. Predefined sample-quality and data-cleaning rules were applied prior to statistical analysis: bloody or cloudy CVL samples were excluded, manganese values below the analytical limit of detection were imputed as 0.01 to permit inclusion in continuous models, and iron and manganese measurements exceeding 3 SD above the overall mean were excluded to reduce the influence of extreme values; the final number of available CVL samples contributing to each comparison is reported as n in the tables. Statistical analyses were performed in IBM SPSS Statistics, version 29 (IBM Corp., Armonk, NY, USA), and continuous variables are presented as mean ± standard deviation (SD). Because participants contributed repeated measurements across visits, longitudinal group comparisons of iron and manganese were performed using linear mixed-effects models with a random intercept for participant to account for within-participant correlation with iron and manganese modeled separately. Primary comparisons between symptomatic culture-positive vaginitis and asymptomatic controls included group as a fixed effect and, where applicable, visit as a categorical fixed effect. We conducted additional subgroup analyses stratified by randomized hydrogel exposure to address the embedded randomized trial. Effect estimates are reported as model-based mean differences (β) with 95% confidence intervals and two-sided p-values; statistical significance was set at *p* < 0.05, and subgroup results were interpreted cautiously without formal adjustment for multiple comparisons.

**Table 1:** The operating parameters of ICP-OES

Parameter	Value
Pump speed (rpm)	15
Sample uptake time (sec)	15
Rinse time (sec)	30
Stabilization time (sec)	20
Nebulizer gas flow (L/min)	0.70
Number of repetitions	3

**Table 2:** The operating parameters and wavelength of ICP-OES

Element	Wavelength (nm)	Observational mode	Reading time (sec)
Fe	238.204	axial	10
Mn	257.610	axial	10

## Results

Eighty women were enrolled in this preliminary dataset of the ongoing clinical trial. The mean age was 36.0 ± 11.8 years, and the mean BMI was 23.1 ± 4.9 kg/m². 19% (*n* = 15) reported a history of vaginal delivery, 11% (*n* = 9) were postmenopausal, and 26% (*n* = 21) had a history of gynecological intervention (Table 3). These descriptors reflect a heterogeneous outpatient cohort with a broad age distribution and normal-range BMI.

**Table 3:** Baseline characteristics of the study cohort

Age (years, mean ± SD)	36 ± 11.8
BMI (kg/m2, mean ± SD)	23.1 ± 4.9
History of vaginal delivery (n, %)	15 (19)
Postmenopausal (n, %)	9 (11)
History of gynecological intervention (n, %)	21 (26)

*N* = 80, values are mean ± SD or n (%). Units: age in years; BMI in kg/m²

Eight women with culture-positive vaginitis and asymptomatic controls were compared using linear mixed-effects models with a random intercept for participant where applicable models were adjusted for visit as a fixed effect. Cervicovaginal lavage Fe concentrations were significantly higher in the vaginitis group than in controls (56.36 ± 24.84, *n* = 70 vs. 44.64 ± 20.11, *n* = 130; Δ = 8.82, 95% CI 0.74 to 16.89, *p* = 0.032; visit-adjusted), while Mn concentrations did not differ between groups (1.42 ± 0.73, *n* = 73 vs. 1.09 ± 0.77, *n* = 127; Δ=−0.08, 95% CI − 0.33 to 0.17, *p* = 0.542; visit-adjusted) (Table 4). In subgroup analyses, among participants who did not receive the zinc-containing hydrogel, Fe remained significantly higher in the vaginitis cohort compared with controls (60.69 ± 26.53, *n* = 27 vs. 47.27 ± 21.87, *n* = 68; Δ = 14.31, 95% CI 0.59 to 28.04, *p* = 0.041; visit-adjusted), whereas Mn showed no significant difference (1.47 ± 0.62, *n* = 28 vs. 1.22 ± 0.74, *n* = 68; Δ=−0.24, 95% CI − 0.61 to 0.13, *p* = 0.209) (Table 5). Among participants who received the hydrogel, Fe concentrations were numerically higher in the vaginitis group than controls but were not statistically significant (53.64 ± 23.62, *n* = 43 vs. 41.75 ± 17.71, *n* = 62; Δ = 7.20, 95% CI − 2.55 to 16.94, *p* = 0.148; visit-adjusted), and Mn concentrations were also comparable (1.39 ± 0.80, *n* = 45 vs. 0.93 ± 0.77, *n* = 59; Δ = 0.11, 95% CI − 0.23 to 0.44, *p* = 0.536; visit-adjusted) (Table 6).

**Table 4:** Compare Fe and Mn levels between vaginitis vs. control

Fe	Vaginitis mean ± SD [*n*]	Control mean ± SD [*n*]	Effect size Δ	95% CI	Direction	*p*-value	Model
	56.36 ± 24.84 [70]	44.64 ± 20.11 [130]	8.82	[0.74, 16.89]	Higher in Vaginitis	0.032	visit-adjusted
Mn	1.42 ± 0.73 [73]	1.09 ± 0.77 [127]	−0.08	[−0.33, 0.17]	Higher in Control	0.542	visit-adjusted

Values are mean ± SD. Units: µg/L. Effect size Δ from linear mixed-effects model with random intercept for participant; visit included as a fixed effect when model convergence allowed

*Abbreviations*: *CVL* Cervicovaginal lavage, *Fe* Iron, *Mn* Manganese

**Table 5:** Compare Fe and Mn levels between vaginitis vs. control (did not use the zinc-containing hydrogel)

Fe	Vaginitis mean ± SD [*n*]	Control mean ± SD [*n*]	Effect size Δ	95% CI	Direction	*p*-value	Model
	60.69 ± 26.53 [27]	47.27 ± 21.87 [68]	14.31	[0.59, 28.04]	Higher in Vaginitis	0.041	visit-adjusted
Mn	1.47 ± 0.62 [28]	1.22 ± 0.74 [68]	−0.24	[−0.61, 0.13]	Higher in Control	0.209	visit-adjusted

**Table 6:** Compare Fe and Mn levels between vaginitis vs. control (used the zinc-containing hydrogel)

Fe	Vaginitis mean ± SD [*n*]	Control mean ± SD [*n*]	Effect size Δ	95% CI	Direction	*p*-value	Model
	53.64 ± 23.62 [43]	41.75 ± 17.71 [62]	7.20	[−2.55, 16.94]	Higher in Vaginitis	0.148	visit-adjusted
Mn	1.39 ± 0.80 [45]	0.93 ± 0.77 [59]	0.11	[−0.23, 0.44]	Higher in Vaginitis	0.536	visit-adjusted

Within the vaginitis cohort, culture-pattern comparisons showed no significant differences in Fe or Mn between bacterial-positive and fungal-positive cultures. In the subgroup that did not receive the hydrogel, Fe did not differ between bacterial-positive and fungal-positive samples (57.40 ± 24.97, *n* = 18 vs. 60.28 ± 32.05, *n* = 4; Δ=−2.96, 95% CI − 30.17 to 24.26, *p* = 0.831) and Mn was similarly comparable (1.44 ± 0.72, *n* = 19 vs. 1.49 ± 0.27, *n* = 5; Δ=−0.03, 95% CI − 0.67 to 0.61, *p* = 0.928); due to convergence constraints these models were not adjusted for visit (Table 7). Among hydrogel users, Fe remained similar between bacterial-positive and fungal-positive cultures (54.92 ± 26.67, *n* = 28 vs. 57.38 ± 16.56, *n* = 4; Δ = 5.15, 95% CI − 21.47 to 31.77, *p* = 0.704; visit-adjusted), and although Mn was numerically higher in fungal-positive samples (2.11 ± 0.55, *n* = 4) compared with bacterial-positive samples (1.30 ± 0.80, *n* = 29), the difference was not statistically significant (Δ=−0.47, 95% CI − 1.20 to 0.26, *p* = 0.207; visit-adjusted) (Table 8). Finally, when stratifying the vaginitis cohort by fungal culture status, Fe concentrations did not differ between fungal culture-negative and fungal culture-positive samples (55.89 ± 25.77, *n* = 46 vs. 57.25 ± 23.46, *n* = 24; Δ=−1.00, 95% CI − 13.35 to 11.35, *p* = 0.874; visit-adjusted), and Mn concentrations were also comparable (1.35 ± 0.76, *n* = 48 vs. 1.54 ± 0.66, *n* = 25; Δ=−0.07, 95% CI − 0.38 to 0.25, *p* = 0.681; visit-adjusted) (Table 9).

**Table 7:** Iron and manganese concentrations in cervicovaginal lavage among women with vaginitis who did not use the zinc-containing hydrogel comparing bacterial-positive with fungal-positive cultures

Fe	Bacterial-positive mean ± SD [*n*]	Fungal-positive mean ± SD [*n*]	Effect size Δ	95% CI	Direction	*p*-value	Model
	57.40 ± 24.97 [18]	60.28 ± 32.05 [4]	−2.96	[−30.17, 24.26]	Higher in Fungal-positive	0.831	unadjusted
Mn	1.44 ± 0.72 [19]	1.49 ± 0.27 [5]	−0.03	[−0.67, 0.61]	Higher in Fungal-positive	0.928	unadjusted

**Table 8:** Iron and manganese concentrations in cervicovaginal lavage among women with vaginitis who used the zinc-containing hydrogel comparing bacterial-positive with fungal-positive cultures

Fe	Bacterial-positive mean ± SD [*n*]	Fungal-positive mean ± SD [*n*]	Effect size Δ	95% CI	Direction	*p*-value	Model
	54.92 ± 26.67 [28]	57.38 ± 16.56 [4]	5.15	[−21.47, 31.77]	Higher in Bacterial-positive	0.704	visit-adjusted
Mn	1.30 ± 0.80 [29]	2.11 ± 0.55 [4]	−0.47	[−1.20, 0.26]	Higher in Fungal-positive	0.207	visit-adjusted

**Table 9:** Iron and manganese concentrations in cervicovaginal lavage in the vaginitis cohort comparing fungal culture-negative cases with fungal-positive cases

Fe	Fungal-negative mean ± SD [*n*]	Fungal-positive mean ± SD [*n*]	Effect size Δ	95% CI	Direction	*p*-value	Model
	55.89 ± 25.77 [46]	57.25 ± 23.46 [24]	−1.00	[−13.35, 11.35]	Higher in Fungal-positive	0.874	visit-adjusted
Mn	1.35 ± 0.76 [48]	1.54 ± 0.66 [25]	−0.07	[−0.38, 0.25]	Higher in Fungal-positive	0.681	visit-adjusted

## Discussion

To our knowledge we are the first to report that vaginal zinc treatment tempers vaginitis-associated iron levels in cervicovaginal lavage. One plausible mechanism is that topical zinc therapy potentially mitigates elevated vaginal iron by restoring epithelial integrity, reducing inflammation and limiting pathogen-driven iron availability. ICP-OES quantifies total elemental burden in CVL, therefore our results reflect total Fe and Mn rather than functional pools. Our findings do not exclude clinically relevant shifts in bioavailable versus protein-bound fractions, redox/speciation states or micro-scale localization during inflammation. Human data on iron and manganese levels in cervicovaginal secretions have not been previously determined. However, several studies have examined how trace elements relate to pregnancy and infections. One research group ran coordinated IVF cohorts and showed that essential and non-essential elements measured in follicular fluid, blood, and urine were linked to ovarian response, embryo development, implantation, and live birth [18–20]. Other pregnancy cohorts connected metals to preterm birth and premature rupture of membranes. Third-trimester urine study highlighted copper as a strong predictor of preterm birth, and a first-trimester blood study found higher barium, chromium, and thallium associated with greater PROM (premature rupture of membrane) risk, partly through changes in the vaginal microbiome [21]. Laboratory and animal work also point to manganese as important at the vaginal surface. In Candida albicans two NRAMP transporters (Smf12 and Smf13) are required for manganese uptake and virulence, and tissues show lower manganese during infection [22]. In a murine model of group B Streptococcus, bacteria with an intact manganese transporter colonized better than transporter-deficient mutants under pressure from the host protein calprotectin [23]. Despite this broad interest, direct measurements of metals in human cervicovaginal fluid have remained limited, and situation-dependent changes are still poorly characterized. In this study, we quantified total iron and manganese concentrations in human cervicovaginal lavage and explored how vaginitis status, culture patterns, and adjunct topical therapy relate to trace-element levels in the vaginal microenvironment. Beyond providing initial reference values for total Fe and Mn, our findings suggest that vaginitis is associated with a measurable shift in cervicovaginal iron, while manganese appears comparatively stable. We observed higher total cervicovaginal iron concentrations in women with culture-positive vaginitis compared with asymptomatic controls, whereas manganese concentrations did not differ between groups. This pattern is biologically plausible, as mucosal inflammation and epithelial disruption can increase local iron through enhanced permeability and release of iron-containing host components. Increased iron availability may also reinforce a pathogen-permissive niche, given central role of Fe in microbial metabolism and virulence. Subgroup analyses suggested that adjunct zinc-containing hydrogel use may attenuate the vaginitis-associated elevation in iron. The vaginitis–control difference was evident among participants who did not use the gel but appeared blunted among those who did. While this observation does not establish causality, it aligns with zinc’s known contributions to epithelial repair and inflammatory modulation, which could indirectly reduce inflammation-driven iron availability at the vaginal surface. Within the vaginitis cohort, iron and manganese concentrations were broadly similar across culture-defined phenotypes. Neither bacterial-positive versus fungal-positive cultures nor fungal culture-negative versus fungal-positive samples showed meaningful differences in Fe or Mn, and small fungal-positive strata limited power for detecting subtle phenotype-specific effects. These results may indicate that total Fe and Mn in lavage are unlikely to serve as practical biomarkers for distinguishing vaginitis etiologies, although they remain informative as reference measures for future mechanistic studies. One of the main strengths of our study is that it is the first to directly quantify iron and manganese concentrations in human cervicovaginal lavage samples, thereby providing novel human data on trace element homeostasis in the vaginal microenvironment. The differentiation of vaginitis phenotypes and the embedded randomized design strengthen internal validity and facilitate comparability with future studies. The limitations of this study include its relatively small sample size, which may have reduced the ability to detect subtle differences. Given the sample size and subdivision into multiple vaginitis phenotypes, the study is primarily powered to detect moderate or larger differences in total Fe and Mn, and smaller effect sizes may not have been observable. Additionally, the dilutional nature of cervicovaginal lavage sampling may introduce variability, as differences in dilution could potentially obscure true concentration changes. The zinc-containing hydrogel used in the parent study may also represent a potential confounding factor, as exogenous zinc exposure could influence local metal handling and thereby affect the interpretation of Fe and Mn measurements. Future studies in larger cohorts should apply standardized dilution control, account for key confounders such as topical zinc exposure, and incorporate host metal-binding and inflammatory markers (e.g., lactoferrin, calprotectin) together with measurements of labile metal fractions to better delineate how nutritional immunity relates to vaginal dysbiosis and symptomatic infection.

## Conclusion

This preliminary analysis suggests that culture-positive vaginitis is associated with higher total cervicovaginal iron concentrations compared with asymptomatic controls, while manganese concentrations remain largely unchanged. Adjunct vaginal zinc treatment may temper vaginitis-associated iron elevation, consistent with a model in which topical zinc supports epithelial restoration and limits inflammation-driven iron availability. Within the vaginitis cohort, total Fe and Mn concentrations did not differ meaningfully across culture-defined phenotypes, indicating limited utility as discriminatory diagnostic biomarkers. These findings provide initial reference values for total cervicovaginal metals and support future studies focused on bioavailable metal pools, metal-binding proteins, and their relationships to symptom dynamics and treatment response.

## Data Availability

The datasets used and/or analyzed during the current study are available from the corresponding author upon reasonable request.

## Abbreviations

BMI: Body Mass Index
CSS: Candida Severity Score
CVL: Cervicovaginal Lavage
Fe: Iron
HNO₃: Nitric Acid
H₂O₂: Hydrogen Peroxide
ICP-OES: Inductively Coupled Plasma Optical Emission Spectrometry
Mn: Manganese
NaCl: Sodium Chloride
PROM: Premature Rupture of Membranes
ROS: Reactive Oxygen Species
SD: Standard Deviation
VSQ-21: Vulvovaginal Symptoms Questionnaire (21-item)
